# Micro RNAs and cancer

**DOI:** 10.3332/ecancer.2010.ed6

**Published:** 2010-04-30

**Authors:** Linda Cairns

**Affiliations:** Science Editor, ecancermedicalscience, European Institute of Oncology, Milan, Italy

The biological processes that lead to cancer and the recent surge of discoveries relating the over-expression of specific micro RNAs (miRNAs) to cancer prognosis, featured prominently in the 101st Annual AACR meeting programme.

MiRNAs are small molecules that regulate gene expression. Initially it was unclear whether altered miRNA expression observed in cancer was a cause or consequence of malignant transformation however numerous studies clearly demonstrated that widespread reduction in miRNA expression does, indeed, promote tumorigenesis.

“The science of miRNAs and related small RNAs will continue to generate new insights into cancer and possible future treatments” said Nobel Laureate Phillip A. Sharp head of the Koch Institute for Integrative Cancer Research at the Massachusetts Institute of Technology, Cambridge, at a press conference presenting some of the miRNAs related talks at the recent 101st AACR conference in Washington DC. One of the presenters at this conference, Sumaiyah Rehman, discussed the effect of a specific microRNA, Mir-21, on trastuzumab (Herceptin) therapy. For Her2/neu positive breast cancer patients, the standard treatment is trastuzumab + chemotherapy but approximately 35% of patients will develop resistance to treatment. Rehman,thinks that the loss of a specific protein PTEN, a tumour suppressor is a key player in the resistance in this group of breast cancers. As miR-21 targets the PTEN mRNA leading to PTEN protein downregulation, the researchers thought that the increased levels of Mir-21 and the reduced levels of PTEN could result in the resistence to trastuzumab. They tested their hypothesis in cell lines which overexpress HER-2 and found that cells with higher levels of Mir-21 had reduced levels of PTEN and were much more resistant to trastuzumab than control cells. These results are supported by results from the clinic which indicated that in Her-2 positive breast cancer patients with higher levels of Mir-21 had a poorer response to Herceptin which also correlated with disease progression.

Eva Hernando also presented results from her laboratory in which they have identified a group of miRNAs specifically associated with brain metastasis from melanoma. Brain matasteses occur in 40%–60% of melanoma patients and the there is no effective treatment. They also found that this group of miRNAs could be already identified in the primary tumour and so identification of this profile at the time of diagnosis may help in deciding effective treatments for these more aggressive tumour types. Also targets of these miRNAs could represent novel therapeutic targets.

Liselle Bovell also discussed data relating a higher expression of a group of miRNAs and poorer prognosis in colorectal cancer patients.

It is now clear that these tiny molecules can have significant effects in cancer progression. The results presented at the recent AACR meeting in Washington represent just a small fraction of the ongoing studies being carried out to try to better understand the biological pathways that are regulated by these tiny molecules and how the information gained can be used in the clinic to develop potentially effective treatments.

